# Patient Attitudes Toward Testicular Prosthesis Placement After Orchiectomy

**DOI:** 10.1177/1557988319861019

**Published:** 2019-07-30

**Authors:** Ashwin Srivatsav, Adithya Balasubramanian, Mohit Butaney, Nannan Thirumavalavan, J. Abram McBride, Jabez Gondokusumo, Alexander W. Pastuszak, Larry Lipshultz

**Affiliations:** 1 Baylor College of Medicine, Houston, TX, USA; 2 Department of Urology, Mayo Clinic, Rochester, MN, USA; 3 Scott Department of Urology, Baylor College of Medicine, Houston, TX, USA; 4 Center for Reproductive Medicine, Baylor College of Medicine, Houston, TX, USA; 5 Division of Urology, Department of Surgery, University of Utah School of Medicine, Salt Lake City, UT, USA

**Keywords:** sexual health, sexuality, urologic surgery, body image, quality of life, general health and wellness

## Abstract

Orchiectomy is the standard of care for many testicular conditions. Testicular prosthesis placement (TPP) can mitigate psychosocial burden, restore self-image, and improve quality of life for patients requiring orchiectomy. Limited data exist regarding patient attitudes and counseling on TPP in the United States. The objective of this study was to characterize patient experiences after TPP, rationale for pursuing/declining TPP, and satisfaction levels.

Patients with a history of urologic conditions warranting orchiectomy were identified and sent an anonymous survey addressing demographics, pre/post counseling, attitudes toward TPP, satisfaction rates, and postoperative complications. Sixteen percent (76/480) of patients completed the survey. Of these, 50.8% (32/63) undergoing orchiectomy were counseled by their surgeon about TPP, and 22.2% (14/63) received a prosthesis. The most common reasons for declining TPP included lack of concern for cosmetic appearance and lack of counseling. Leading reasons for pursuing TPP included improving self-confidence and cosmetic appearance. Although 71% (10/14) of patients were satisfied with TPP, they did highlight areas for improvement. Twenty percent (2/10) felt their implant was too high, 60% (6/10) felt their implant was too firm, 10% (1/10) endorsed discomfort during sex, and 30% (3/10) felt that TPP did not match their size expectations. Despite these findings, 71% (10/14) reported that they would have TPP again and 79% (11/14) would recommend TPP to others.

TPP improves body image and quality of life following orchiectomy. Provider counseling plays an important role in influencing a patient’s decision to undergo TPP. Areas of improvement include implant positioning and more effective replication of testicular consistency.

Orchiectomy is the standard of care for a range of benign and malignant testicular conditions (Mohammed, Yassin, Hendry, & Walker, 2015). Young men undergoing orchiectomy endorse feelings of unease, shame, or humiliation following testicle removal due to a perceived loss of masculinity (Skoogh et al., 2011). Testicular prosthesis placement (TPP) is available to patients following orchiectomy in order to minimize psychological distress and restore quality of life and self-esteem (Turek & Master, 2004). TPP is an option for severe testicular atrophy, cryptorchidism, or any noninfectious condition requiring orchiectomy, including malignancy and emergent injury such as torsion or testicular rupture (Bodiwala, Summerton, & Terry, 2007; Dieckmann et al., 2015).

Testicular prosthesis quality has improved considerably since the first documented use of a vitallium alloy implant in 1941 (Girsdansky & Newman, 1941). Patients noted dissatisfaction with the vitallium implant’s cold, metallic sensation, and felt it inaccurately represented testicular consistency (Girsdansky & Newman, 1941). Newer prostheses were initially comprised of silicone implants filled with gel in an effort to use physiologically inactive materials and more precisely replicate the natural consistency of the testicle (Lattimer, Vakili, Smith, & Morishima, 1973). Gel-filled implants were replaced by saline-filled prostheses in 1995 as a precautionary measure after discovery that many silicone breast implants had leaked into surrounding connective tissues, causing harm (Robinson, Bradley, Wilson, & Fisher, 1995). Following the transition to saline-filled prosthesis, TPP cemented itself as a safe and effective reconstructive option after testicular removal (Turek & Master, 2004).

While TPP is indicated for patients undergoing orchiectomy with various benign and malignant conditions, few studies in the United States have investigated TPP counseling and patient satisfaction. TPP-oriented studies have primarily been conducted in Europe (Adshead, Khoubehi, Wood, & Rustin, 2001). Only two prior studies in the United States have investigated patient satisfaction with TPP in an oncologic context (Clifford et al., 2018; Nichols et al., 2019). Though much more common, mastectomy and breast implant surgery are comparable to orchiectomy and TPP. A recent analysis of trends in breast reconstruction demonstrated that as of 2014, 43.3% of women undergoing mastectomy undergo breast reconstruction (Ilonzo, Tsang, Tsantes, Estabrook, & Thu Ma, 2017). By contrast, only 15.7% percent of men undergoing orchiectomy receive a testicular prosthesis (Mohammed, Yassin, Hendry, & Walker, 2015).

Little is known about efforts to improve and standardize the counseling that surgeons provide to patients who are eligible for TPP. Further investigation into patient perspectives about TPP in the United States is required, given that attitudes toward orchiectomy and TPP, to some degree, are culturally driven (Saab, Noureddine, Abu-Saad Huijer, & Dejong, 2014). With the hypothesis that patients who undergo orchiectomy and are adequately counseled on TPP have high rates of satisfaction, the purpose of this study was to characterize patient experiences with TPP, specifically regarding patient counseling prior to orchiectomy, rationale for pursuing or declining TPP, and overall patient satisfaction with the implant.

## Methods

Patients presenting to Baylor College of Medicine between May 2009 and February 2018 with ICD codes signifying history of a urologic condition that warranted radical or simple orchiectomy were identified in the urology department at Baylor College of Medicine via Institutional Review Board (IRB) approved protocol H-42501 (Investigation of the Clinical Use and Patient-Reported Outcomes for Testicular Prosthesis Use after Orchiectomy). All patients were identified based on billing codes CPT 54520, 54530, 54535, and 54660. Patients were required to be over 18 and have an accessible e-mail address available in the electronic medical record. An anonymous 27 question survey was designed and electronically disseminated through email using Survey Monkey (San Mateo, California, USA) to assess patient demographics, counseling about TPP, attitudes toward testicular implants, satisfaction rates, and postoperative complications. A cover letter was sent electronically notifying patients that if they completed the survey they were providing consent that the results could be used anonymously for this research study. Respondents were further queried about specific indications for their orchiectomy, decisions to pursue/decline implants, rationale for their decisions, and whether they would recommend TPP to others. Questions used in the survey are presented in Supplementary Figure S1. Results were anonymously submitted and analyzed. Descriptive analyses were used to report pertinent variables.

## Results

Sixteen percent (77/480) of patients responded to the survey, and 13% (63/480) patients were included in the final analysis. The 3% (14/480) of patients excluded from analysis stated that they did not undergo orchiectomy. Two respondents indicated that they underwent orchiectomy as part of a gender reassignment procedure and four declined to provide a reason for the procedure. Demographic characteristics of the cohort are presented in Table 1. Most (41%, 26/63) patients identified as “White/Caucasian” followed closely by Hispanic/Latino (27%, 17/63).

**Table 1.** Patient Demographics.

Variable	Number of respondents (*n* = 63) *N* (%)
**Age:**	
0–20	**4 (6.3%)**
21–25	**7 (11.1%)**
26–30	**10 (15.9%)**
31–35	**11 (17.5%)**
36–40	**4 (6.3%)**
41–45	**4 (6.3%)**
46–50	**6 (9.5%)**
50+	**10 (15.9%)**
Not given	**7 (11.1%)**
**Race:**	
Hispanic/Latino	**17 (27.0%)**
White/Caucasian	**26 (41.3%)**
Black/African American	**5 (7.9%)**
Asian/Pacific Islander	**2 (3.2%)**
Decline answer	**13 (20.6%)**
**Household Income**:	
<$10,000	**2 (3.2%)**
$10,001–$20,000	**4 (6.3%)**
$20,001–$40,000	**8 (12.7%)**
$40,001–$70,000	**8 (12.7%)**
$70,001–$90,000	**3 (4.8%)**
>$90,000	**18 (28.6%)**
Decline answer	**20 (31.7%)**
**Education**:	
8^th^ grade or less	**1 (1.5%)**
Some high school	**3 (4.8%)**
High school graduate (or GED)	**7 (11.1%)**
Some college	**20 (31.7%)**
College graduate	**11 (17.5%)**
Graduate or doctoral coursework (or degrees)	**10 (15.9%)**
Decline answer	**11 (17.5%)**

Patients’ reasons for undergoing TPP are characterized in Table 2. A majority of patients (73%, 46/63) underwent orchiectomy for malignancy or risk of malignancy. Only 27% (17/63) underwent orchiectomy for benign indications. Testicular trauma, injury, and infection were the leading benign indications.

**Table 2.** Characterization of Reasons for Orchiectomy, TPP, and Satisfaction.

Variable	Number of respondents *N* (%)
**Reasons for orchiectomy:**	
Malignancy/malignancy risk	**46 (73%)**
Torsion	**1 (2%)**
Trauma/injury	**4 (6%)**
Infection	**2 (3%)**
Pain	**2 (3%)**
Other	**8 (13%)**
**Most common reasons for receiving TPP**	
Self-confidence	**4 (28.6%)**
Cosmetic appearance to others	**7 (50%)**
Cosmetic appearance to self	**3 (21.4%)**
**Most common reasons for not receiving TPP**	
Cosmetic appearance to others not a concern	**20 (40.8%)**
Cosmetic appearance to self not a concern	**19 (38.8%)**
TP not offered by surgeon	**18 (36.7%)**
Complication risk	**10 (20.4%)**
Cost	**7 (14.3%)**
TP not covered by insurance	**5 (10.2%)**
TP offered but unavailable	**1 (2%)**
**Satisfied with TPP**	
Yes	**10 (71%)**
No	**4 (29%)**
**Would receive implant again if necessary**	
Yes	**10 (72%)**
No	**2 (14%)**
No answer given	**2 (14%)**
**Would recommend TPP to others**	
Yes	**11 (79%)**
No	**1 (7%)**
No answer given	**2 (14%)**

Of the respondents, 50.8% (32/63) of men surveyed indicated that they were counseled by their surgeon or another physician about the possibility of receiving TPP at the time of orchiectomy, whereas 36.5% (23/63) of men were not counseled on TPP, and 12.7% (8/63) declined to answer. Of the respondents, 22.2% (14/63) elected to receive TPP following orchiectomy. Of these 14 men, 64.3% (9/14) received the implant at the time of orchiectomy, while 35.7% (5/14) received the implant as a separate procedure. Further, 92.9% (13/14) indicated that they had been counseled regarding TPP at the time of orchiectomy.

Of the men who elected to undergo TPP, 50% (7/14) stated that concern with cosmetic appearance to others was their primary reason for electing to undergo the procedure. Other reasons for undergoing TPP included increased self-confidence (29%, 4/14) and concern with cosmetic appearance to self (21.4%, 3/14). Men cited a broad range of reasons for declining TPP. Of the 49 patients who declined TPP, 40.8% (20/49) reported that cosmetic appearance to others was not a concern, and 38.8% (19/49) reported that cosmetic appearance to self was not a concern. Surprisingly, 36.7% (18/49) reported that TPP was not offered by the surgeon. Of these 18 patients, 67% (12/18) did not receive counseling in the setting of testicular cancer. The remaining 33% (6/18) did not receive counseling regarding TPP in the setting of a range of urgent or emergent conditions including testicular torsion, trauma/injury, pain, or infection. Patients also reported that they declined TPP due to concerns about complications (20.4%, 10/49), cost (14.3%, 7/49), and lack of insurance (10.2%, 5/49).

Patient satisfaction with TPP is also summarized in Table 2; 71.4% (10/14) patients who received TPP were satisfied with the decision and would undergo TPP again if necessary, and 78.6% (11/14) said they would recommend TPP to other men undergoing orchiectomy. Only 28.6% (4/14) men indicated that they were dissatisfied with the implant. Notably, none of the patients who reported dissatisfaction with the implant identified any specific issues or complications with their implants and declined to elaborate on the source of their dissatisfaction.

Although most patients were satisfied with TPP, several identified areas in which their implant can be improved; 20% (2/10) felt the implant was too high in the scrotum and 60% (6/10) felt that the implant was too firm. Only 10% (1/10) noted that the implant was uncomfortable during sexual activity as a result of it being too large, and 10% (1/10) noted that the implant was too small.

Men who did not undergo TPP were asked if they regretted their decision; 83.7% (41/49) men responded to this question, with 26.8% (11/41) stating that they regretted not receiving a TPP. Notably, 63.6% (7/11) of these men reported that they were not counseled on TPP by their surgeon.

## Discussion

An association between testicular removal and psychological feelings of shame and loss has been previously established (Skoogh et al., 2011). Following orchiectomy, more than 50% patients surveyed noted either missing the removed testicle or feeling unease as a result of its absence (Skoogh et al., 2011). Long-term survivors of testicular cancer further noted negative changes in their own body image (Rossen, Pedersen, Zachariae, & Von Der Maase, 2012). Ofman supported this association by detailing the psychological burden placed on young men following diagnosis and treatment of testicular cancer (Ofman, 1995). Improved body image and self-esteem is common in patients who received an implant, and previous work by Adshead et al. noted that 73% of men surveyed were happy with their implant (Adshead et al., 2001). Further studies done on patient satisfaction after TPP by Yossepowitch et al. demonstrated that 28% of men rated their satisfaction with their implant as excellent and another 45% rated it as good (Yossepowitch, Aviv, Wainchwaig, & Baniel, 2011). Though few studies originating in the United States have focused on characterizing patient experiences with TPP satisfaction following orchiectomy, Clifford et al. reported that 82.5% of men rated their implant as excellent, 87.5% would receive it again, and 92.5% of men found the implant to be comfortable (Clifford et al., 2018). Only two prior U.S. studies have investigated patient satisfaction with TPP following orchiectomy (Clifford et al., 2018; Nichols et al., 2019). As a result, the present work focused on characterizing patient experiences with TPP at the time of orchiectomy.

Testicular cancer patients asked about counseling regarding TPP expressed concerns that counseling was too abbreviated and not comprehensive (Dieckmann et al., 2015). In the present study, 18 out of 49 (37%) men who did not receive TPP indicated that they had not received any form of counseling at time of orchiectomy. Furthermore, 7 out of 11 (63.6%) men who endorsed regret over not receiving TPP reported that they were not counseled on TPP by their surgeon. This lack of counseling persisted across benign and malignant conditions requiring orchiectomy, suggesting that providers are inadequately counseling patients in various clinical contexts. This reinforces the findings of Nichols et al., who reported in a questionnaire-based study of genital satisfaction in men who had undergone orchiectomy that 42% of men who did not receive TPP were not offered an implant by their surgeon (Nichols et al., 2019). Importantly, most who underwent TPP in the present study had been counseled about the implant at time of surgery.

No standardized protocol exists to counsel patients about TPP at the time of orchiectomy. The results of this study suggest that surgeons tend to overlook counseling about TPP in emergent settings, likely due to the acuity of the presenting condition. Although TPP is an effective and safe standalone procedure, most urologists would advocate for simultaneous TPP at time of orchiectomy (Mohammed et al., 2015). Consequently, comprehensive counseling about TPP at the time of orchiectomy may ensure greater satisfaction and restoration of self-esteem. Several patients cited perceived risks associated with TPP as a reason for rejecting the implant. Complications following TPP are low and include extrusion (8%), scrotal contraction (3%–5%), pain (1%–3%), hematoma (0.3%–3%), and infection (0.6%–2%; Marshall, 1986). Patients should be counseled that TPP is considered a safe and effective intervention, with low complication rates over the last 50 years (Lakshmanan & Docimo, 1997). Furthermore, Turek and Master demonstrated that modern saline-filled prostheses are safe, well tolerated, and improve quality of life (Turek & Master, 2004). Additionally despite these risks, a 2014 study of 904 men who underwent radical orchiectomy demonstrated that 236 received a prosthesis and only 1 out of 236 (0.4%) required a prosthesis removal (Robinson, Tait, Clarke, & Ramani 2016). These studies therefore reinforce that counseling by surgeons can improve patients’ awareness about TPP safety and eliminate such concerns.

Patients cited that cost or lack of insurance coverage prevented them from pursuing TPP. Although many commercial insurance companies anecdotally cover the cost of TPP following orchiectomy, no prior studies have formally investigated whether coverages vary between malignant and benign conditions. In a broader context, lack of information on insurance coverage for urologic reconstruction makes it difficult for patients to feel comfortable pursuing TPP. By contrast, the Women’s Health and Cancer Rights Act of 1999 compelled all insurance policies to cover immediate breast reconstruction (IBR) after mastectomy. Subsequently, rates of IBR increased by 4.2-fold in women covered by Medicaid, 2.9-fold in women covered by Medicare, and 2.6-fold in privately insured patients (Yang et al., 2013). Despite the usage of native tissue for some breast reconstruction procedures (which is not an option for TPP), studies on insurance coverage and satisfaction with breast implants continue to be more prevalent than studies on insurance and satisfaction with testicular prostheses (Catanzariti, Polito, & Polito, 2016). Future work oriented toward elucidating TPP cost and insurance coverage should be undertaken in order to enable surgeons to counsel patients regarding TPP more effectively.

Studies done by Adshead et al., Bodiwala et al., and Skoogh et al. reported that psychological feelings of loss and shame are reduced in patients who received an implant (Adshead et al., 2001; Bodiwala et al., 2007; Skoogh et al., 2011). This study further demonstrates that patients with TPP report high satisfaction rates and would recommend it to others. Several patients indicated areas for improvement, primarily citing concerns about implant firmness and positioning within the scrotum. These results are consistent with prior work that identified similar concerns about implant materials and placement (Dieckmann et al., 2015), which together could help guide manufacturers to improve prosthesis design by more accurately replicating natural testicular consistency and shape. Improvements in prostheses as well as counseling by physicians can empower patients and improve overall satisfaction with TPP.

The present work has several limitations which warrant further discussion. A nonvalidated anonymous self-reported survey was utilized to query patients. Studies employing this methodology suffer from varying degrees of respondent bias and omission, which are augmented by the retrospective nature of the study. Furthermore, the single center survey distributed via email yielded only 63 patients who underwent orchiectomy. Of these 63, only 14 underwent TPP, which limits the generalizability of the results. It also limits the degree to which statistical comparisons can be used to make meaningful inferences regarding relationships between orchiectomy indications, counseling, and satisfaction. While a higher number of responses would be desirable, this rate is comparable to other studies that have investigated patient satisfaction with TPP. Despite these limitations, the present work highlights areas for improvement in care of men requiring orchiectomy.

## Conclusions

The present study observed high satisfaction and positive attitudes toward TPP among patients receiving testicular prostheses following orchiectomy. Patients noted increased self-confidence and decreased concern about appearance as key rationales for pursuing TPP, reinforcing the utility of TPP in alleviating psychosocial burden associated with testicular removal. This study identified that lack of counseling by surgeons was a key barrier for patients eligible for TPP. Patients’ concerns about complications associated with TPP was also a common reason for declining an implant, despite a strong safety record and low risk occurrence. Future work is required to improve counseling for patients undergoing orchiectomy inclusive of highlighting the low complication rates and high safety of TPP, given that testicular implants can restore self-esteem following orchiectomy.
